# Mobile clinics for antiretroviral therapy in rural Mozambique

**DOI:** 10.2471/BLT.13.129478

**Published:** 2014-06-19

**Authors:** Troy D Moon, Tito Jequicene, Meridith Blevins, Eurico José, Julie R Lankford, C William Wester, Martina C Fuchs, Sten H Vermund

**Affiliations:** a Vanderbilt Institute for Global Health, 2525 West End Avenue, Suite 750, Nashville, Tennessee 37203, United States of America (USA).; b Friends in Global Health, Limited Liability Corporation, Quelimane, Mozambique.; c Real Medicine Foundation, Los Angeles, California, USA.

## Abstract

**Problem:**

Despite seven years of investment from the President's Emergency Plan For AIDS Relief (PEPFAR), the expansion of human immunodeficiency virus (HIV)-related services continues to challenge Mozambique’s health-care infrastructure, especially in the country’s rural regions.

**Approach:**

In 2012, as part of a national acceleration plan for HIV care and treatment, Namacurra district employed a mobile clinic strategy to provide temporary manpower and physical space to expand services at four rural peripheral clinics. This paper describes the strategy deployed, the uptake of services and the key lessons learnt in the first 18 months of implementation.

**Local setting:**

In 2012, Namacurra´s adult population was estimated to be 125 425, and of those 15 803 were estimated to be HIV infected. Although there is consistent government support of antiretroviral therapy (ART) programmes, national coverage remains low, with less than 15% of those eligible having received ART by December 2012.

**Relevant changes:**

Between April 2012 and September 2013, Namacurra district enrolled 4832 new patients into HIV care and treatment. By using the mobile clinic strategy for ART expansion, the district was able to expand provision of ART from two to six (of a desired seven) clinics by September 2013.

**Lessons learnt:**

Mobile clinic strategies could rapidly expand HIV care and treatment in under-funded settings in ways that both build local capacity and are sustainable for local health systems. The clinics best serve as a transition to improved capacity at fixed-site services.

## Résumé

**Problème:**

Malgré les 7 années d'investissement du Plan d'urgence du Président des États-Unis d'Amérique pour la lutte contre le SIDA (PEPFAR), l'expansion des services de lutte contre le virus de l'immunodéficience humaine (VIH) est toujours un défi pour les infrastructures de soins de santé du Mozambique, en particulier dans les régions rurales du pays.

**Approche:**

En 2012, dans le cadre d'un plan d'accélération pour les soins et le traitement contre le VIH, le district de Namacurra a utilisé une stratégie de dispensaires mobiles pour fournir une main-d'œuvre temporaire et de l'espace physique afin d'étendre les services de lutte contre le VIH à 4 dispensaires périphériques ruraux. Cet article décrit la stratégie déployée, l'assimilation de ces services et les principaux enseignements tirés au cours des 18 premiers mois de mise en œuvre.

**Environnement local:**

En 2012, la population adulte de Namacurra était estimée à 125 425 personnes, et on estimait que 15 803 d'entre elles étaient infectées par le VIH. Malgré un soutien constant des programmes de traitement antirétroviral (TAR) de la part du gouvernement, la couverture nationale reste faible, avec moins de 15% des personnes éligibles ayant reçu un TAR en décembre 2012.

**Changements significatifs:**

Entre avril 2012 et septembre 2013, le district de Namacurra a inscrit 4832 nouveaux patients au programme de soins et de traitement contre le VIH. En utilisant la stratégie de dispensaires mobiles pour étendre le TAR, le district a pu développer la fourniture de TAR de 2 à 6 dispensaires (pour un nombre souhaité de 7) en septembre 2013.

**Leçons tirées:**

Les stratégies de dispensaires mobiles pourraient rapidement étendre les soins et les traitements contre le VIH dans les endroits sous-financés par des moyens qui renforcent les capacités locales et qui sont durables pour les systèmes de santé locaux. Les dispensaires servent au mieux de transition vers l'amélioration des capacités dans les services de site fixe.

## Resumen

**Situación:**

A pesar de los siete años de inversión del Plan de Emergencia del Presidente de los Estados Unidos de América para luchar contra el SIDA (PEPFAR), la expansión de los servicios relacionados con el virus de la inmunodeficiencia humana (VIH) continúa suponiendo un desafío para la infraestructura sanitaria de Mozambique, especialmente en las regiones rurales del país.

**Enfoque:**

En 2012, como parte de un plan de aceleración nacional para el cuidado y tratamiento del VIH, el distrito de Namacurra empleó una estrategia clínica móvil para suministrar mano de obra y espacio físico temporales para expandir los servicios a cuatro clínicas periféricas rurales. Este documento describe la estrategia empleada, la comprensión de servicios y las lecciones clave aprendidas en los primeros 18 meses de implementación.

**Marco regional:**

En 2012, la población adulta estimada de Namacurra era de 125 425 y de estas personas se estimaba que 15 803 estaban infectadas con el VIH. A pesar de haber un constante apoyo gubernamental de los programas de tratamiento antirretroviral (TAR), la cobertura nacional sigue siendo baja, menos de un 15 % de las personas seleccionadas habían recibido TAR antes de diciembre de 2012.

**Cambios importantes:**

Entre abril de 2012 y septiembre de 2013, el distrito de Namacurra inscribió a 4832 nuevos pacientes en el cuidado y tratamiento del VIH. Mediante el uso de la estrategia de la clínica móvil para la expansión de TAR, el distrito pudo expandir las provisiones de TAR de dos a seis clínicas (de las siete deseadas) antes de septiembre de 2013.

**Lecciones aprendidas:**

Las estrategias de las clínicas móviles podrían expandir rápidamente el cuidado y tratamiento del VIH en situaciones de poca financiación de forma que crean capacidad local y sean sostenibles para los sistemas sanitarios locales. Las clínicas sirven de la mejor manera como transición a la capacidad mejorada en servicios en lugares fijos.

## ملخص

**المشكلة:**

رغم مرور سبعة أعوام من الاستثمار على خطة الرئيس الطارئة للإغاثة من مرض الإيدز (PEPFAR)، ما زال التوسع في الخدمات ذات الصلة بفيروس العوز المناعي البشري يشكل تحدياً للبنية الأساسية للرعاية الصحية في موزامبيق، لا سيما في المناطق الريفية من البلد.

**الأسلوب:**

في عام 2012، استخدمت مقاطعة ناماكورا استراتيجية العيادات المتنقلة، كجزء من خطة التسريع الوطنية لرعاية فيروس العوز المناعي البشري وعلاجه، لتوفير القوة العاملة المؤقتة والمساحة المادية لتوسيع الخدمات في أربع عيادات ريفية. وتتناول هذه الورقة بالوصف الاستراتيجية التي تم نشرها والاستفادة من الخدمات والدروس الرئيسية المستفادة في أول 18 شهراً من التنفيذ.

**المواقع المحلية:**

في عام 2012، كان عدد السكان في سن البلوغ في ناماكورا وفق التقديرات 125425 نسمة، وكان من بينهم 15803 شخصاً مصابين بعدوى فيروس العوز المناعي البشري. ورغم الدعم المتسق المقدم من الحكومة لبرامج العلاج بمضادات الفيروسات القهقرية، لا تزال التغطية الوطنية منخفضة، حيث حصل أقل من 15 % فقط من المؤهلين على العلاج بمضادات الفيروسات القهقرية بحلول كانون الأول/ ديسمبر 2012.

**التغيّرات ذات الصلة:**

بين نيسان/ أبريل 2012 وأيلول/ سبتمبر 2013 سجلت مقاطعة ناماكورا 4832 مريضاً جديداً في رعاية فيروس العوز المناعي البشري وعلاجه. وعن طريق استخدام استراتيجية العيادات المتنقلة لتوسيع العلاج بمضادات الفيروسات القهقرية، تمكنت المقاطعة من توسيع نطاق توفير العلاج بمضادات الفيروسات القهقرية من عيادتين إلى ست عيادات (من أصل سبع عيادات مرجوة) بحلول أيلول/ سبتمبر 2013.

**الدروس المستفادة:**

تتمتع استراتيجيات العيادات المتنقلة بالقدرة على توسيع نطاق رعاية فيروس العوز المناعي البشري وعلاجه على نحو سريع في المناطق منخفضة التمويل بطرق يمكنها بناء القدرة المحلية وتتمتع بالاستدامة بالنسبة للنظم الصحية المحلية. وتمثل العيادات تحولاً إلى تحسين القدرة في خدمات المواقع الثابتة.

## 摘要

**问题:**

尽管获得总统防治艾滋病紧急救援计划（PEPFAR）七年的投资，莫桑比克的医疗基础设施依然面临着扩大艾滋病毒（HIV）相关服务的挑战，在该国的农村地区尤其如此。

**方法:**

在2012年，作为全国性HIV护理和治疗加速计划的组成部分，纳玛库拉（Namacurra）地区采用移动诊所策略来提供临时人力和物理空间，以扩大四个农村外围诊所的服务。本文介绍了在开始18个月的实施过程中部署的战略、提供的服务和吸取的关键经验教训。

**当地状况:**

2012年，纳玛库拉成年人口估计为125425人，其中估计有15803人受到艾滋病毒的感染。虽然始终有抗逆转录病毒疗法（ART）项目的政府支持，但是这种治疗的全国覆盖率仍然很低，到2012年12月，有资格获得这种治疗的人群中仅有不到15%的人接受了ART的治疗。

**相关变化:**

在2012年4月至2013年9月，纳玛库拉地区招募了4832名新病人接受艾滋病护理和治疗。通过使用移动诊所战略进行ART扩展，该地区能够将ART治疗的提供点从两个诊所增加到2013年9月的六个（预期需要七个）。

**经验教训:**

流动诊所策略可以迅速扩大资金短缺地区艾滋病的护理和治疗，其采取的方式既有利于建设本地能力，对当地卫生系统也是可持续的。这些诊所是改进固定网点服务能力的最好过渡形式。

## Резюме

**Проблема:**

Несмотря на инвестиции, которые в течение семи лет выделялись в рамках Чрезвычайного плана Президента США по борьбе со СПИДом (PEPFAR), распространение услуг по борьбе с вирусом иммунодефицита человека (ВИЧ) продолжает оставаться проблемой для инфраструктуры здравоохранения Мозамбика, особенно в сельских регионах страны.

**Подход:**

В 2012 году в рамках Национального плана по активизации ухода и лечения ВИЧ-инфекции в районе Намакурра была использована стратегия передвижных клиник, которые предоставляли временный персонал и физическое пространство для расширения ассортимента услуг в четырех сельских периферийных клиниках. В этой статье описывается использованная стратегия, потребление услуг и главные уроки, извлеченные в ходе первых 18 месяцев реализации стратегии.

**Местные условия:**

В 2012 году взрослое население Намакурры оценивалось в 125 425 человек, из которых 15 803 человека, по оценкам, были инфицированы ВИЧ. Несмотря на постоянную государственную поддержку программ антиретровирусной терапии (АРТ), охват населения на национальном уровне остается низким, и менее чем 15% населения, имеющего право на АРТ, получили лечение к декабрю 2012 года.

**Осуществленные перемены:**

В период с апреля 2012 года по сентябрь 2013 года в районе Намакурра 4832 новых пациента были зачислены для участия в программе ухода и лечения ВИЧ. Используя стратегию передвижных клиник для расширения охвата АРТ, район смог расширить предоставление АРТ с двух до шести (из желаемых семи) клиник к сентябрю 2013 года.

**Выводы:**

Стратегии передвижных клиник могут быстро расширить охват услугами ухода и лечения ВИЧ в условиях недостаточного финансирования таким путем, какой позволит нарастить местный потенциал, и являются рациональными для местных систем здравоохранения. Такие клиники являются наилучшим средством перехода к улучшенному потенциалу оказания услуг на базе стационарных учреждений.

## Introduction

The high burden of human immunodeficiency virus (HIV) infections, acquired immunodeficiency syndrome, tuberculosis, malaria and under-five mortality in sub-Saharan Africa has driven the global community to explore both new and old ideas to alleviate these problems. The roll-out of mobile clinics to broaden health-service coverage received considerable attention in the 1960s and 70s, but was not adopted on a permanent basis due to logistic challenges.^1^^,^^2^ Today, in Mozambique, mobile clinic strategies are again being rolled-out to increase coverage with antiretroviral therapy (ART). Here, we report on 18 months of experience using one mobile clinic in Zambézia Province, Mozambique.

### Local setting

In 2009, Mozambique had an HIV prevalence of 11.5%, with 1.4 million people being HIV positive.^3^ Although there is consistent government support of ART programmes, national coverage remains low, with less than15% of those eligible having received ART by December 2012.^4^ ART scale-up has posed challenges for Mozambique’s under-capacitated health-care infrastructure.^5^^-^^13^ If Mozambique is to achieve a national target of 80% ART coverage by 2015, then scale-up efforts must continue to be strengthened.

The magnitude of the HIV epidemic is especially evident in Zambézia Province, which is Mozambique’s second largest province and home to 4 million people.^3^^,^^9^^,^^10^ Zambézia has low literacy, poor maternal and child health indices, high rates of tuberculosis and malaria, high malnutrition, and comparatively low adult and paediatric ART coverage.^9^^,^^10^^,^^12^^-^^14^

**Fig. 1:**
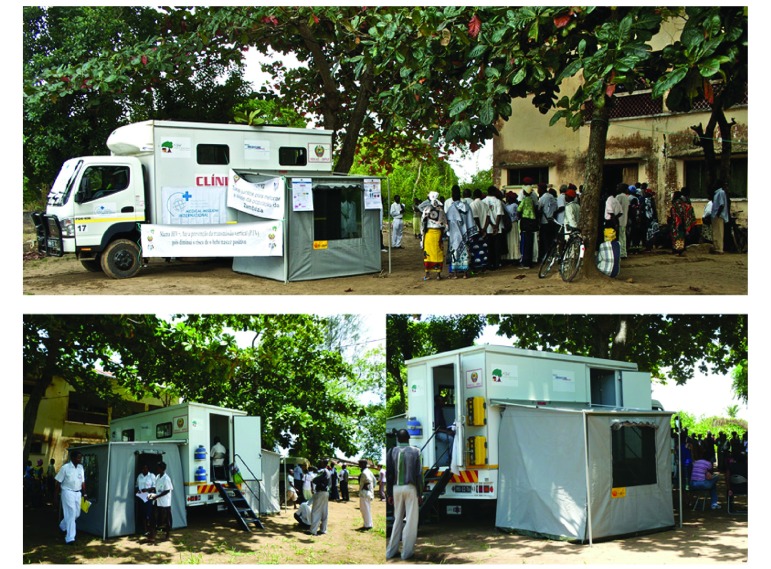
A mobile clinic, Mozambique, 2013

Friends in Global Health, which is affiliated with the Vanderbilt Institute for Global Health, has been providing technical assistance for HIV in Zambézia since 2007. In 2009, Friends in Global Health partnered with the Real Medicine Foundation to deploy a mobile clinic built on a four-wheel drive truck that was initially used for short-term HIV counselling and testing campaigns and for emergency response following natural disasters. The clinic is 6.3 m in length and able to operate in poor road conditions; it comprises two rooms inside the vehicle cage – one equipped for clinical consultations and the other as a pharmacy, with two side tents that provide space for HIV counselling and testing (Fig. 1).

Courtesy of the Friends in Global Health community health team

In early 2012, Mozambique’s Ministry of Health, in conjunction with its partners from the President’s Emergency Plan for AIDS Relief (PEPFAR), initiated an acceleration plan for further scale-up of the country’s HIV programmes, to overcome the low ART coverage. Since April 2012, we have used funds from the Real Medicine Foundation and PEPFAR to expand ART via the mobile clinic in the district of Namacurra, Zambézia Province. This district has approximately 125 425 adults, 15 803 of whom are infected with HIV, based on an estimated 2009 HIV prevalence of 12.6%.^3^

## Approach

The Ministry of Health acceleration plan included expansion of ART services in Namacurra from two clinics in January 2013 to seven by the end of 2013. Before expansion, many clinics required more and better-trained staff and building rehabilitation, presenting a bottleneck to scale-up of services. To achieve service initiation within the 2013 timeframe, strategies were designed for using the mobile clinic to enable rapid expansion.

At first, the mobile clinic acts to reinforce the fixed clinic, providing temporary space for services while necessary changes at the facility are completed. Services offered by the mobile clinic include HIV care and treatment; point-of-care measurements of CD4+ T lymphocyte count and haemoglobin; activities to address retention in care before and after ART initiation; tuberculosis diagnosis and care; management of malaria, diarrhoea and malnutrition; and targeted maternal and child health care. The mobile clinic staff serves as direct health-care providers and they also train and mentor staff of the fixed clinic through a learn-by-doing approach in which mobile clinic and fixed-site clinicians work side-by-side in the care and treatment of HIV positive patients, including pregnant woman and exposed children. This mentored training also focuses on aspects of HIV service functionality, such as patient chart documentation and laboratory and pharmacy logistical needs. Additionally, they actively collaborate with community organizations to ensure that patients have access to HIV-prevention services, including educational materials, condoms, referral for male circumcision, psychosocial support, nutrition counselling, support for orphans and vulnerable children, and home-based care if needed.

The mobile clinic is staffed by a non-physician health officer, a maternal child health nurse, a pharmacy technician, a lay counsellor, and a driver, all hired by Friends in Global Health. It functions from Monday to Thursday, and alternates weekly between two fixed clinics, thus spending 8 days per month at each site.

## Relevant changes

As of 31 March 2012, Namacurra offered HIV services at only two of its 10 clinics (Namacurra Capital and Macuze), and reported 2146 patients actively enrolled in HIV care. In April 2012, two peripheral clinics – Mixixine and Malei – were chosen to receive initial mobile clinic support. When the clinics were considered to be self-sufficient, the mobile clinic moved from Mixixine to Furquia in February 2013 and from Malei to Mbaua in August 2013. Between April 2012 and September 2013, 4832 new HIV patients were enrolled in six clinics; 1223 of them were enrolled at the four clinics that received active support from the mobile clinic (Table 1).

**Table 1:** Human immunodeficiency virus care and treatment, Namacurra District, Mozambique, April 2012 to September 2013

Site	Period for mobile clinic support	No. of patients active in care or treatment before mobile clinic implementation^a^	No. of newly enrolled patients in care or treatment^b^		
			With mobile clinic present	After departure of mobile clinic	Total
**Fixed health-facility**					
Namacurra Capital	No mobile clinic	1421	NA	NA	2209
Macuze	No mobile clinic	725	NA	NA	1155
**Mobile clinic**					
Mixixine	April 2012 to February 2013	0	360	235	595
Malei	April 2012 to August 2013	0	423	10	433
Furquia	February 2013 to present	0	360^c^	NA	360
Mbaua	August 2013 to present	0	80^d^	NA	80
**Total no.**		**2146**	**1223**	**245**	**4832**

NA: not applicable.

^a^ As of 1 April 2012.

^b^ April 2012 to September 2013.

^c^ Data were obtained between February and September 2013.

^d^ Data were obtained between August and September 2013.

### Start-up process

At each site, HIV services were launched with a health fair, to encourage community acceptance. During the fair, community leaders were invited to speak about the arrival of HIV services and the importance of being tested, initiation of treatment and adherence. Community theatre groups performed skits about HIV and other health issues; voluntary counselling and testing was offered; and nutrition education was shared.

### Laboratory testing

Laboratory testing capacity in Zambézia Province continues to be a limitation for efficient patient care. For Namacurra Capital and Macuze (i.e. ART sites not supported by the mobile clinic), samples for CD4 counts, haematology and biochemistry are transported daily to the district capital laboratory. However, with the initiation of the ART expansion plan, this system for transport of samples was unable to meet the increased demand. In response, the mobile clinic was equipped with a point-of-care CD4 machine, a haemoglobinometer and rapid tests for HIV, malaria and syphilis, so that tests could be run on-site.

Training on phlebotomy and interpretation of laboratory results was provided through a learn-by-doing approach towards the collection of samples and the running of CD4, haemoglobin and various other rapid tests. This mentored training was running throughout the time the mobile clinic remained at the fixed-site. Additionally, joint planning exercises were performed to incorporate the fixed-site clinic into the laboratory sample transport system, thus ensuring continuity of services after the mobile clinic moved on.

### Logistic support for pharmaceuticals

The mobile clinic arrived at the site each day stocked with the necessary antiretroviral medications, tuberculosis medications and medications for common opportunistic infections. All other medications used at the fixed clinic were supplied through the Ministry of Health’s routine channels for drug distribution. The mobile clinic pharmacist provided mentored training on the appropriate use of these medications, documentation and calculation of future stock needs. This ensured that, by the time the mobile clinic moved on to the next site, the fixed clinic had been incorporated into the provincial HIV drug supply network.

### Counselling and support

The lay counsellor was responsible for performing voluntary counselling and testing, enrolling patients into the HIV services, and linking patients with community organizations to ensure delivery of additional services, as appropriate.

## Lessons learnt

The provision of space and personnel through the mobile clinic allowed the district of Namacurra to instantly start expanding the programme to high-priority peripheral clinics. Overall, we feel that this strategy was successful in achieving the goal of rapid HIV service expansion in Namacurra; however, there were notable challenges. The key lesson learnt about programme start-up was the importance of having an open collaborative partnership with district health authorities and with local leaders from the communities surrounding the clinics. Also fundamental from the beginning was engagement with health authorities to map out the resources needed for successfully transitioning activities from the mobile clinic staff to the district clinic staff.

The ART acceleration plan established for the province was quite ambitious and authorities were under significant pressure to expand as quickly as possible. This resulted in the mobile clinic leaving one of its first facilities too hastily. Plans to ensure continuity for pharmacy services, laboratory transport, and monitoring and evaluation were not finalized before departure, resulting in a 1–2 month period of medication stock-outs and limited laboratory support. In response, a checklist was developed to determine whether a site had everything in place to be able to function alone. This checklist was used at each of the subsequent facilities supported by the mobile clinic and greatly improved the transition to local control of the programme (Box 1).

Box 1 **Summary of main lessons learnt**

- The mobile clinic strategy enabled the rapid start-up of HIV care and treatment at fixed-site clinics while the clinics were being renovated for HIV services and their staff were being trained.
- Early engagement of health authorities to map out the resources needed for fixed clinics to function independently, as well as engagement of community leaders to facilitate community acceptance of the mobile clinic strategy, were fundamental to programme success.
- Use of mobile clinic staff as mentors to the local clinic staff enabled the transfer of skills and the rapid exit of the mobile clinic personnel, allowing the mobile clinic to quickly move to new sites.

HIV: human immunodeficiency virus.

## Conclusion

By using the mobile clinic strategy for ART expansion, Namacurra was able to expand provision of HIV care services from two to six (of a desired seven) clinics by September 2013. Following this pilot phase, in June 2013, Friends in Global Health started to deploy two additional mobile clinics in a further two districts of Zambézia Province, using the same strategy that had been employed in Namacurra. Scale-up is currently underway in provinces across the country. Long-term PEPFAR funding is not guaranteed; nevertheless, this strategy will assist the Ministry of Health’s expansion plan for HIV services in the short-term, and should require support for only a few years.

Our experience reflects the realities of severe resource constraints in one of the world´s poorest nations. Manpower shortages and infrastructure limitations constrain more rapid expansion of chronic disease care for those infected with HIV. In the context of these challenges, deployment of a mobile clinic created a short-term solution, enabling services to be provided while the fixed clinics were refurbished and staff trained.
